# Effects of pH on steam explosion extraction of acetylated galactoglucomannan from Norway spruce

**DOI:** 10.1186/s13068-018-1300-z

**Published:** 2018-11-09

**Authors:** Leszek Michalak, Svein Halvor Knutsen, Ida Aarum, Bjørge Westereng

**Affiliations:** 1 Faculty of Chemistry, Biotechnology and Food Science, Norwegian University of Life Sciences, Ås, Norway; 2 Nofima, Norwegian Institute of Food, Fishery and Aquaculture Research, PB 210, 1431 Ås, Norway

**Keywords:** Steam explosion, PH control, Norway spruce, Mannan, Galactoglucomannan, Acetylation, Hemicellulose, Hydrothermal extraction

## Abstract

**Background:**

Acetylated galactoglucomannan (AcGGM) is a complex hemicellulose found in softwoods such as Norway spruce (*Picea abies*). AcGGM has a large potential as a biorefinery feedstock and source of oligosaccharides for high-value industrial applications. Steam explosion is an effective method for extraction of carbohydrates from plant biomass. Increasing the reaction pH reduces the combined severity ($R^{\prime}_{0}$) of treatment, affecting yields and properties of extracted oligosaccharides. In this study, steam explosion was used to extract oligosaccharides from Norway spruce wood chips soaked with sodium citrate and potassium phosphate buffers with pH of 4.0–7.0. Yields, monosaccharide composition of released oligosaccharides and biomass residue, their acetate content and composition of their lignin fraction were examined to determine the impact of steam explosion buffering on the extraction of softwood hemicellulose.

**Results:**

Reducing the severity of steam explosion resulted in lower yields, although the extracted oligosaccharides had a higher degree of polymerization. Higher buffering pH also resulted in a higher fraction of xylan in the extracted oligos. Oligosaccharides extracted in buffers of pH > 5.0 were deacetylated. Buffering leads to a removal of acetylations from both the extracted oligosaccharides and the hemicellulose in the residual biomass. Treatment of the residual biomass with a GH5 family mannanase from *Aspergillus nidulans* was not able to improve the AcGGM yields. No hydroxymethylfurfural formation, a decomposition product from hexoses, was observed in samples soaked with buffers at pH higher than 4.0.

**Conclusions:**

Buffering the steam explosion reactions proved to be an effective way to reduce the combined severity ($R^{\prime}_{0}$) and produce a wide range of products from the same feedstock at the same physical conditions. The results highlight the impact of chemical autohydrolysis of hemicellulose by acetic acid released from the biomass in hydrothermal pretreatments. Lower combined severity results in products with a lower degree of acetylation of both the extracted oligosaccharides and residual biomass. Decrease in severity appears not to be the result of reduced acetate release, but rather a result of inhibited autohydrolysis by the released acetate. Based on the results presented, the optimal soaking pH for fine-tuning properties of extracted AcGGM is below 5.0.

**Electronic supplementary material:**

The online version of this article (10.1186/s13068-018-1300-z) contains supplementary material, which is available to authorized users.

## Background

Steam explosion (SE) is an effective and scalable method for solubilizing hemicellulose from plant biomass, applicable to a wide range of biorefinery feedstocks. SE extraction was successfully used as pretreatment for production of biogas from hay [1], sugarcane bagasse [2] and corn stover [3], birchwood [4] as well as the production of ethanol from spruce bark [5] and many other platform chemicals from a wide range of lignocellulose feedstocks [6].

Steam explosion combines hydrothermal treatment of biomass with defibration by a rapid release of pressure at the end of the process. These two processes are independent of each other, and results comparable with SE have been obtained by hydrothermal treatment with a mechanical refining step, as long as the treatment severity was the same [7]. In the course of the hydrothermal pretreatment, a major part of the hemicellulose and lignin present in the secondary cell wall lamellae is separated from the adjacent cellulose microfibrils and becomes water soluble [8]. At the same time, some of the acetate naturally linked to the xylan and mannan in the lignocellulose is released and contributes to the autohydrolysis of biomass. Release of acetic acid is the reason for the low pH usually seen in the SE product slurry. Properties of SE treated material depend on a range of factors, the most important being the residence time and temperature in the vessel. Impact of temperature on the material is described by the severity factor $R_{0} = {\text{e}}^{{(T_{{{ \exp } }} - 100)/14.75}}$ [9]. A combined severity factor $R^{\prime}_{0} = \left( {10^{{ - {\text{pH}}}} } \right) \times (t \times {\text{e}}^{{(T_{{{ \exp } }} - 100)/14.75}} )$ [10] was developed to include the contribution of H^+^ to the hydrolysis process. This combined severity factor was previously used to predict and compare the severities of treatments where pH, rather than temperature or residence time, was the variable [11, 12]. Mitigating the severity of pretreatment by controlling pH is a potential means of fine-tuning the products.

A number of factors besides temperature and residence time also play a role, such as the biomass particle size and the rate of steam and liquid diffusion through the particle, the ratio of solids to liquid loaded into the SE vessel and the chemicals brought in from upstream processing stages. During SE treatment, acetylated hemicellulose releases acetic acid, which decreases the pH and facilitates chemical hydrolysis of polysaccharides. Acetate-mediated autohydrolysis depends on the diffusion of liquid through the biomass particles [13]. Diffusion rate depends on the particle size and the surface-to-volume ratio. The final pH of the product slurry after hydrothermal pretreatment depends on the composition of the liquid fraction, its amount and buffering capacity. The intricacies of hemicellulose breakdown in hydrothermal pretreatment and difficulties in the analysis of the process are brilliantly explained by Rissanen et al. [13].

For inclusion in microbial fermentation, conditions are usually selected with the aim of highest possible breakdown of biomass, while keeping the formation of chemicals inhibitory to enzymatic hydrolysis or fermentation to a minimum [14, 15]. In the literature, pertaining SE and pretreatments fermentability and end-product yields are often selected as the main evaluation criteria, favoring high severity conditions often using acids or sulfates as additives [6, 16]. These high severity conditions yield oligosaccharides with low degree of polymerization (DP), low degree of acetylation (DA) and fewer branchings, which require fewer enzymes for hydrolysis to monosaccharides. For GGM, this means a partial or complete deacetylation and removal of galactose side chains. In contemporary biorefining focused on production of higher value chemicals such as food and feed ingredients, nutraceuticals [17] or hydrocolloids [18], controlled extraction conditions yielding high molecular mass and high complexity can be a more attractive pretreatment option. With the right enzyme toolbox, further tailoring and breakdown into constituent monomers is easy to achieve, while synthesis of highly branched and decorated polysaccharides in large amounts is almost impossible. Obtaining more complex hemicelluloses is of interest for several reasons: more complex products with novel physicochemical properties open doors to new applications; higher complexity may improve selectivity in microbial degradation [19] and increase the biodiversity of gut microbiomes when used as prebiotics. More complex oligosaccharides that more closely resemble in vivo hemicellulose would also make attractive substrates for studying activity of carbohydrate active enzymes.

Galactoglucomannan (GGM) is the main hemicellulose in Norway spruce (*Picea abies*). It is a complex hemicellulose consisting of a backbone of β-(1→4)-d-Man*p* and β-(1→4)-d-Glc*p* residues with α-(1→6)-d-Gal*p* branches, prevalently attached to the Man*p*, and to a lesser extent on Glc*p* [19]. An estimated 30% of the d-Man*p* residues of spruce GGM  are 2-*O*-, 3-*O*- and 6-*O*-acetylated, as well as 4-*O*-acetylated in the non-reducing ends of oligosaccharides [19]. Acetylation of spruce mannan is a particularly important feature, since it affects the accessibility of mannans to microbes and the physicochemical properties of mannans in solution. At the same time, release of acetylations from hemicellulose and hydrolysis of polysaccharides by the released acetate is a crucial process for the solubilization of hemicellulose [8].

In this study, SE extraction was carried out with pH control resulting in a mitigation of treatment severity. Six experimental conditions at five pH levels as well as a control sample using water only were used for SE to yield significantly different oligosaccharides in the extract. The relationship between the combined severity factor and the product composition was evaluated by assessing the yields, apparent DP, oligosaccharide acetylation, monosaccharide composition of products and biomass residue, MALDI-ToF MS analysis of extracted oligosaccharides, NMR analysis of lignin released and analysis of susceptibility of biomass residue to treatment with a GH5 mannanase.

## Results and discussion

**Fig. 1:**
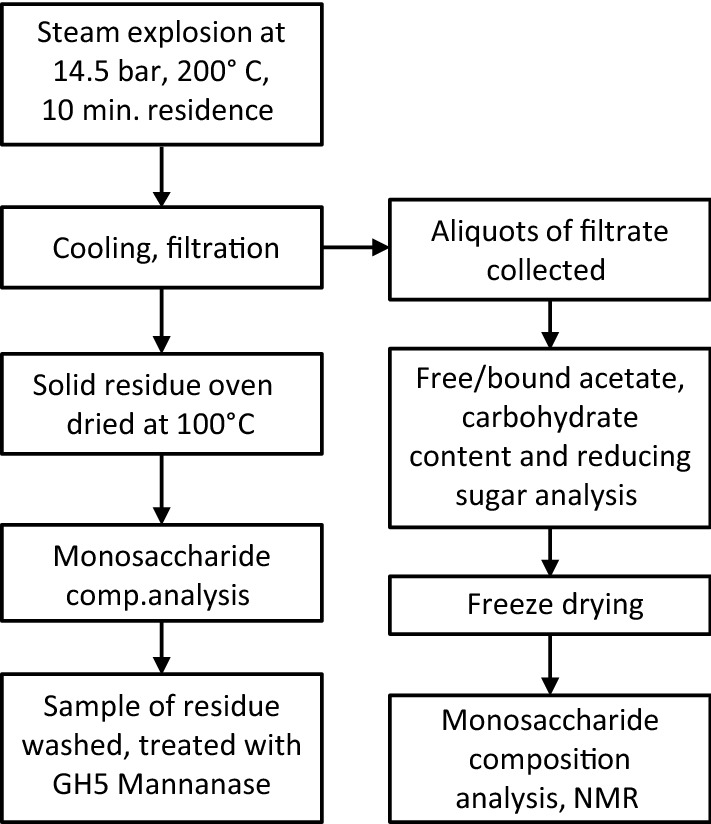
Flowchart of sample treatment and analyses carried out. The steam exploded wood chips were transferred from the collection vessel to plastic buckets and allowed to cool, and pH measurements were taken once the slurry reached room temperature. Water was then added to aid extraction. Samples were mixed and transferred to funnels laid with Whatman B1 filters. Aliquots of this filtrate were used for quantification of acetate content, total carbohydrate content and reducing sugars. Samples of the filtrate were freeze-dried and used for monosaccharide composition analysis and analysis of lignin by NMR. SE wood retained by the filters was dried at 100 °C for 36–48 h, until steady weight was reached. Samples of dried extracted wood were used for monosaccharide composition and enzymatic hydrolysis

A detailed description of sample handling and analysis pipeline is illustrated in the flowchart (Fig. 1). Citrate- and phosphate-based buffers were selected due to their respective buffer ranges and temperature stability. In all samples except the citrate pH 4.0, the pH has dropped after SE due to release of acetate from the wood (Table 1). Higher buffer concentrations would be necessary to keep the post-SE pH exactly as the soaking buffers; however, this would cause more interference with downstream analysis. The range of buffers resulted in combined severities ranging from 0.004 to 0.519 in the buffer controlled samples. Non-buffered controls had the highest $R^{\prime}_{0}$ at 1.68–1.75. The wide range of calculated $R^{\prime}_{0}$ is entirely attributable to the buffered conditions, since other conditions in the reaction were the same. The large difference in $R^{\prime}_{0}$ between the buffered samples illustrates the room for adjustment and possibility for fine-tuning granted by pH controlled extractions.

**Table 1:** Sample treatments, slurry pH after steam explosion and the combined severity factors calculated as in [11] which determine severities based on the pH after the treatment

Buffer	Average pH	St. dev.	Average combined severity $R^{\prime}_{0}$	St. dev.	Man:Glc:Galratio	Bound acetate µmol/mg carbohydrate
MilliQ H_2_O control	3.7	0.009	1.707	0.037	1.88:1:0.28	0.30
0.5 M citrate pH 4.0	4.2	0.009	0.511	0.011	2.71:1:0.39	0.31
0.5 M citrate pH 5.0	5.0	0.041	0.092	0.009	1.64:1:0.51	0.11
0.5 M citrate pH 6.0	5.6	0.012	0.024	0.001	0.67:1:0.53	n.d.
1 M phosphate pH 6.0	5.3	0.045	0.042	0.004	1.39:1:0.23	n.d.
1 M phosphate pH 6.5	5.9	0.017	0.010	0.000	0.19:1:0.11	n.d.
1 M phosphate pH 7.0	6.3	0.031	0.004	0.000	0.23:1:0.16	n.d.

In all samples buffering the SE reaction has resulted in final pH higher (pH 4.2–6.3) than that of the control samples (average pH 3.7). 0.5 M citrate and 1 M phosphate at pH 6.0 resulted in different final pH, highlighting the difference in the buffering capacity between citrate and phosphate

### Yields and composition of extracted hemicellulose

**Fig. 2:**
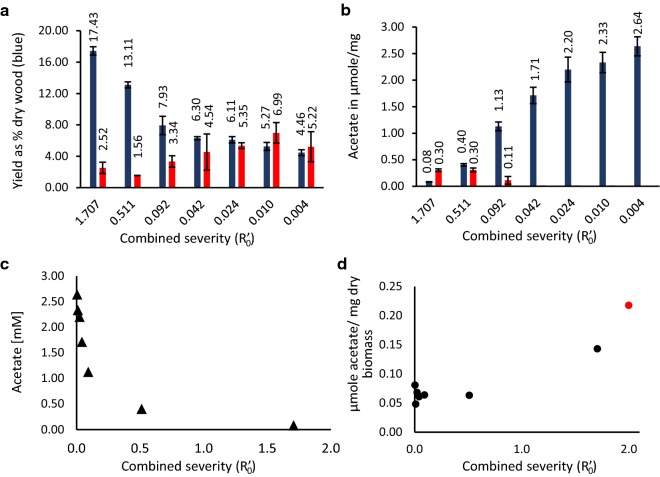
**a** Percentage yields of total carbohydrates (blue bars) from dry wood mass, and the average DP of extracted oligosaccharides (red bars), error bars show standard deviation between technical replicates. **b** Bar chart of acetate in filtrate (blue bars), and acetate released from the oligosaccharides in solution (red bars) after KOH treatment. Error bars indicate the standard deviation between technical replicates. **c** Scatterplot of acetate content of filtered samples at the various severities. **d** Scatterplot of acetate content of dried biomass residue, dry wood raw material in red

Higher severity treatment yielded higher amounts of solubilized carbohydrates, with the highest yield of 17.4% average based on dry wood weight for the non-buffered samples (Fig. 2a). Yields dropped for the buffered samples, with only the citrate pH 4.0 among the buffered samples (average $R^{\prime}_{0}$ = 0.511) being close to the non-buffered sample (13.1% average yields). The total yield of soluble carbohydrates dropped rapidly with the decreasing $R^{\prime}_{0}$ although the yields remained over 4% (4.4% for the potassium phosphate pH 7.0 buffered samples, $R^{\prime}_{0}$ = 0.0045). Yields from the three least severe treatments (sodium citrate pH 6.0, average $R^{\prime}_{0}$ = 0.0241; and potassium phosphate pH 6.5 and 7.0, $R^{\prime}_{0}$ = 0.0104 and $R^{\prime}_{0}$ = 0.0045, respectively) shift very slightly (6.1% for citrate pH 6.0, 5.2% and 4.4% for phosphate pH 6.5 and 7.0, respectively) despite a considerable drop in the $R^{\prime}_{0}$. This decrease in efficiency with increasing pH was attributed to reaction pH being higher than the p*K*_a_ of acetic acid (4.76). Under these conditions, the reactivity of acetic acid and its contribution to autohydrolysis of hemicellulose are markedly decreased. Characteristics of products from these low severity treatments illustrate a baseline for extraction in a SE reaction with a minor contribution of autohydrolysis. The extracts approximate the products of an extraction with steam and temperature only (Additional file 1: Figure S1).

In order to assess yields as well as the degree of hemicellulose breakdown occurring during extraction, total carbohydrate content of each sample was determined using the phenol–sulfuric acid method of Dubois [20]. Concentrations of reducing sugars were estimated by Miller’s dinitrosalycilic acid assay [21]. For comparison of severity effects on the estimated length of oligosaccharides in the soluble fraction, the ratio of total carbohydrates to reducing sugars was used as an approximation for the DP of the solubilized oligosaccharides (Fig. 2a). Comparison of yields and DP of extracted oligosaccharides shows the increase in average DP (from 2.52 at $R^{\prime}_{0}$ = 1.707 to 5.22 at $R^{\prime}_{0}$ = 0.004), accompanied by a reduction in yields (decrease from 17.4% of dry wood weight at $R^{\prime}_{0}$ = 1.707 to 4.4% at $R^{\prime}_{0}$ = 0.004). An overview of oligosaccharide length and sample composition is presented in MALDI-ToF MS spectra (Additional file 2: Figure S2). Multiple oligosaccharides with *m/z* over 1000 are present in all samples, despite the comparison of total to reducing sugars indicating the average DP range to be between 2.52 (control) and 6.99 (potassium phosphate pH 6.5). This apparent discrepancy is due to the fact that MALDI-ToF was not able to detect monosaccharides and cleary visualize the oligosaccharides < 750 *m/z* due to high background from the salts and other contaminants in the samples.

### Composition of extracted hemicellulose

**Fig. 3:**
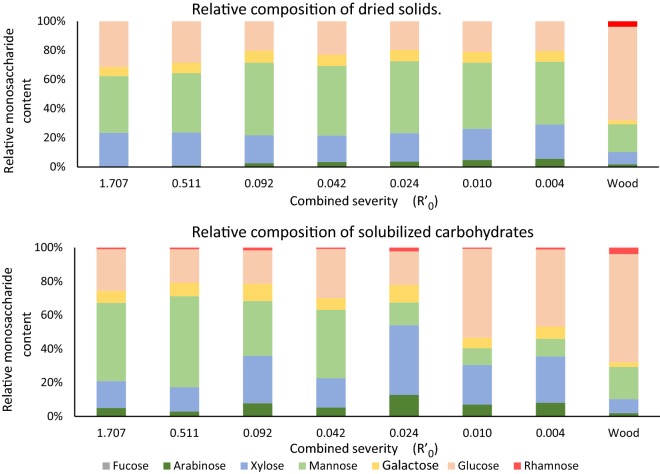
Top: relative monosaccharide composition of carbohydrates in the dried, washed solids. Bottom: relative monosaccharide composition of carbohydrates in aqueous extracts of steam exploded wood. The composition of untreated spruce chips raw material (wood) is provided for comparison

Beside the yields and apparent DP, buffering the SE reaction had an impact on the composition of extracted oligosaccharides. A comparison of the monosaccharide composition of all samples and the extracted wood is summarized in Fig. 3 and Additional file 3: Tables S2 and S3. At lower severity, more xylooligosaccharides were released, with only the citrate buffered and control samples yielding GGM as the predominant hemicellulose. No rhamnose was detected in the dried solids biomass residue after any treatment. Loss of arabinose in the high severity samples can be attributed to hydrolysis observed previously in low pH extractions [22]. In samples buffered with pH 6.0, 6.5 and 7.0 phosphate, the relative content of mannose in the solubilized carbohydrate fraction was several times lower than that of xylose (Fig. 3, Additional file 3: Table S2). Galactose content and the apparent Gal/Man ratio have increased with decreasing severity, although we were unable to ascertain whether the galactose was bound to GGM oligosaccharides or was present as monosaccharides resulting from debranching of GGM in the cell wall. The decrease in efficiency of extraction over the wide range of severities is apparent in Fig. 3, and the content of hemicellulose left in dried biomass residue increases with the decrease in combined severity.

**Fig. 4:**
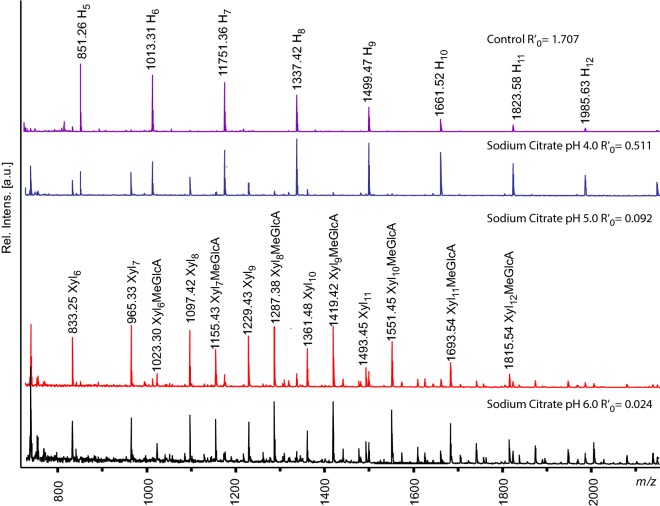
MALDI-ToF MS Spectra of extract samples deacetylated with NaOH. In the control and sodium citrate pH 4.0 samples, GGM peaks are the main components, with small xylooligosaccharide peaks alongside GGM in the sodium citrate pH 4.0. In sodium citrate pH 5.0 and 6.0 the dominant peaks are the xylooligosaccharides and methylglucuronic acids. Xyl, xylose; H, hexose; MeGlcUA, methylglucuronic acid; Ac, acetylation

The gradual shift from extraction of AcGGM toward xylan and glucuronoxylan is illustrated in Fig. 4. In order to clear the MALDI-ToF spectrum and avoid ambiguity of *m/z* assignment (such as in the case of peak 1097 *m/z*, which appears in the hexose and pentose series), aliquots of the extracts were deacetylated by adding 100 mM NaOH. The control sample and sodium citrate pH 4.0 samples spectra contain predominantly hexose peaks, while xylooligosaccharide peaks are dominant in the spectra of citrate pH 5.0 and 6.0 samples.

**Fig. 5:**
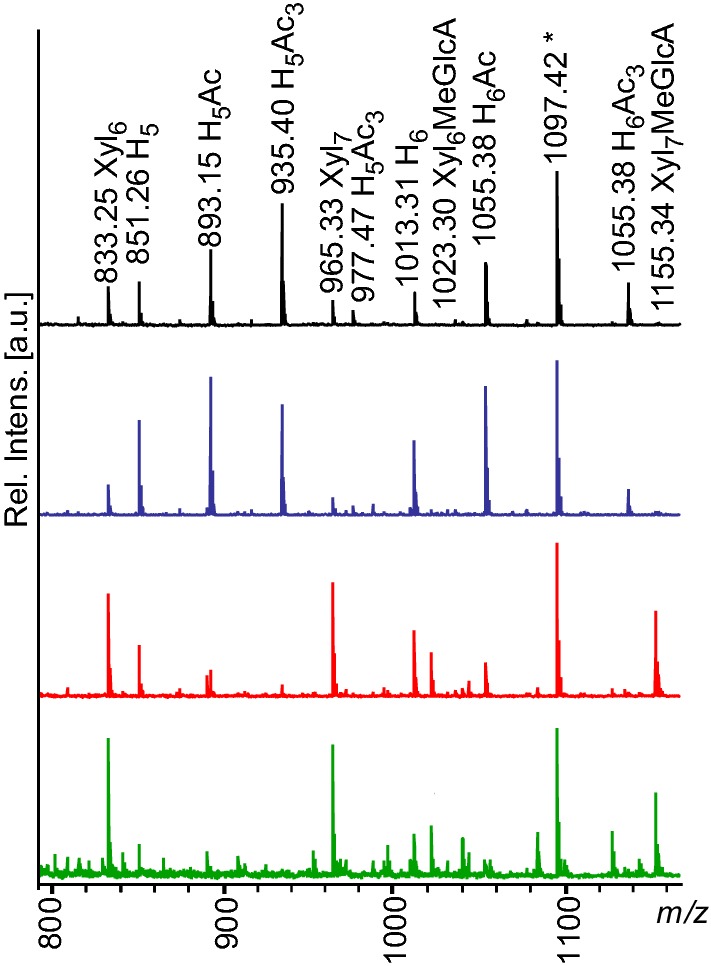
MALDI-TOF MS spectra of extracted oligosaccharides from the buffer control (black), sodium citrate pH 4.0 (blue), sodium citrate pH 5.0 (red), sodium citrate pH 6.0 (green). Peak labeled 1097.42* is either a double acetylated mannohexose or non-acetylated octapentose. Xyl, xylose; H, hexose; MeGlcUA, methylglucuronic acid; Ac, acetylation

Extraction buffered with sodium citrate at pH 4.0 produced the highest relative content of GGM in the filtrate (Fig. 3) and with the highest degree of acetylation of extracts (Figs. 2b and 5). The apparent increase in relative mannan content in soluble fraction of citrate pH 4.0 buffered samples ($R^{\prime}_{0}$ = 0.511) comes at a reduction of yield from 17.3 to 13.1% compared to the control sample (Fig. 2a). The corresponding dried biomass residue samples have a very similar monosaccharide distribution: 37.56% mannose and 6.01% galactose for the control sample residue, 39.51% mannose and 6.92% galactose for citrate pH 4.0 (Additional file 3: Table S2). SE with citrate pH 4.0 buffering appears more selective toward mannan, while the unbuffered control had a higher overall efficiency.

The Man:Glc:Gal ratio (Table 1) is an indication of complexity of yielded mannooligosaccharides. In high severity hydrothermal extraction, the α-(1→6)-d-Gal*p* branchings of GGM are cleaved off [23]. For the Norway Spruce (*Picea abies*), the Man:Glc:Gal ratios reported in the literature range from 4:1:0.1 to 3.8:1:0.4 [19, 24]. The ratio varies based on the wood and extraction methods. When the GGM constituent ratios are considered, buffering with citrate at pH 5.0 has yielded the best results, nearly doubling the galactose content of the extracted oligos from control samples (Table 1). The ratios were 1.88:1:0.28 Man/Glc/Gal in the control samples and 1.64:1:0.51 in the citrate pH 5.0. At the same time, citrate at pH 5.0 increased the apparent DP of the oligosaccharides from 2.52 to 3.34 (Fig. 2a). The improvement in Man:Glc:Gal ratio was accompanied with a pronounced decrease in yield (7.93% for citrate pH 5.0) and the mannose content of the extract (32.55% for citrate pH 5.0 vs 46.41% for control). Citrate pH 5.0 extracts contained 28.09% xylose, nearly twice as much as the control (15.94% for xylose) (Additional file 2: Figure S2 and Additional file 3: Table S2 and S3) and had nearly three times lower degree of acetylation (Fig. 2b).

### Acetate content of soluble fractions

Acetate content in the filtrate decreased quickly with increasing severity (Fig. 2c). The same trend was apparent in analysis of acetate content in biomass residue. Biomass from buffered samples contained between 0.06 and 0.04 µmol of acetate per mg of biomass (Fig. 2d), while the control samples contained 0.14 µmol of acetate per mg. Dried biomass from control samples retained 65.8% of the acetate measured in wood raw material (0.14 µmol vs 0.22 µmol of acetate per mg). Since it is difficult to estimate the factual DP of oligosaccharide products, acetylation values were calculated as µmole of acetate per mg of solubilized carbohydrates.

In the severity range between the control samples and the samples buffered with sodium citrate pH 6.0, hemicellulose peaks seen in MALDI-ToF MS gradually became deacetylated (Fig. 5). The relative intensities of peaks corresponding to acetylated mannooligosaccharides indicate that the highest content of acetylated mannooligos was extracted in the control sample. Acetylated mannooligos are the majority of peaks in the control and sodium citrate pH 4.0 samples and disappear in sodium citrate pH 6.0 samples.

Aliquots of the aqueous extracts were treated with KOH to deacetylate the oligosaccharides in solution. KOH treatment removed the acetylations on oligos in solution and allowed for comparison between the free acetate and bound acetate. Only the citrate pH 4.0, 5.0, and the control samples contained appreciable amounts of acetate bound to carbohydrates (Fig. 2b, Table 1). Despite the fact that high pH and low severity conditions yield more acetate per mg of released hemicellulose, the acetate was present free in solution. Whether this occurred as a result of pH in the SE vessel or occurred during storage of the sample (since buffer solution is still present) is unclear. From previous, unpublished experimental results at the same scale, as well as pilot scale where over 700 kg of spruce was processed, we know that storage at the control sample pH (3.6–4.0) did not cause a deacetylation even at ambient temperatures, for 2–4 weeks.

Acetylation of extracted oligosaccharides is a characteristic crucial for their physicochemical properties. The DA affects water solubility, susceptibility to enzymatic hydrolysis and availability as a carbon source for microbes. Release of acetate during hydrothermal pretreatment is one of the mechanisms of cell wall breakdown, and a decrease in severity would be expected to correlate with a decrease in the acetate released and in the amounts of acetate bound to oligosaccharides. This was, however, not the case as more acetate was released with higher buffer pH. This may be due to de-esterification which is accelerated at higher pHs [25].

### Enzymatic treatment of solid residue

**Fig. 6:**
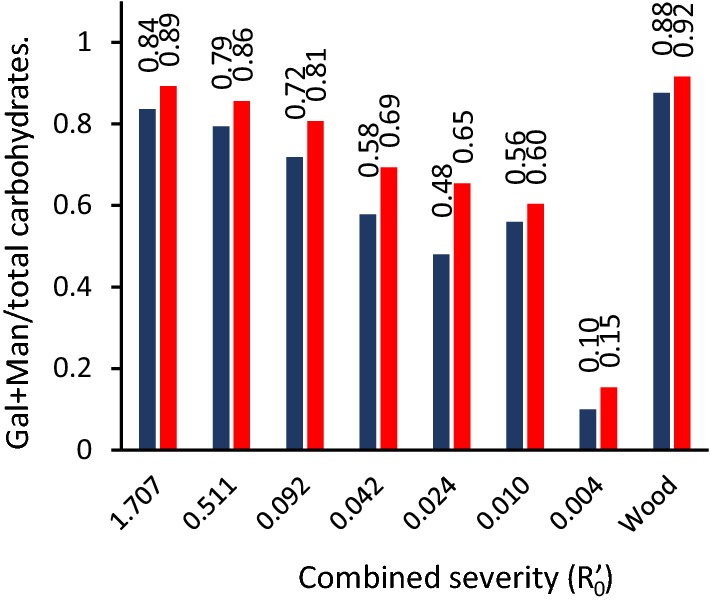
Content of galactose and mannose as a fraction of total carbohydrates extracted with GH5 mannanase treatment. Red bars represent the Gal + Man fraction in mannanase treated samples; blue bars represent control samples with no enzyme

Enzymatic hydrolysis was tested as a means to assist the release of hemicellulose from wood treated with SE in conditions of inhibited autohydrolysis. Samples of dried residual biomass were treated with a GH5 family endomannanase from *Aspergillus nidulans* [26] to find out whether severity of SE had an effect on the availability of hemicellulose in the steam exploded wood to hydrolytic enzymes. Even at low combined severity, the hemicellulose matrix is exposed to extreme conditions and undergoes defibration in the pressure release. These conditions were hypothesized to open the secondary cell wall matrix and render the hemicellulose more accessible to mannanases. GH5 family mannanases have been shown to be more efficient on less acetylated substrates [27], and since a large part of the acetate was removed in the steam explosion, it was hypothesized that a hydrolytic enzyme could to a larger extent access the residual mannan and thus improve the yields of mannooligosaccharides. However, mannanase treatment of dried residual biomass from SE did not release appreciable amounts of mannooligosaccharides, indicating that mannan in the biomass residue remains largely inaccessible to hydrolytic enzymes, regardless of the material being acetylated (high severity) or non-acetylated (low severity). While there was an apparent effect of the mannanase when the relative content of carbohydrates in enzyme treatment solution was analyzed (Fig. 6), the only observable effect was a slight increase in the combined galactose and mannose fraction of the released oligosaccharides as compared to the control sample incubated at the same conditions in buffer without the enzyme. Enzymatic treatment with this enzyme was not a viable means of improving the yields of low severity SE.

### NMR analysis of lignin content in the solubilized fraction

**Fig. 7:**
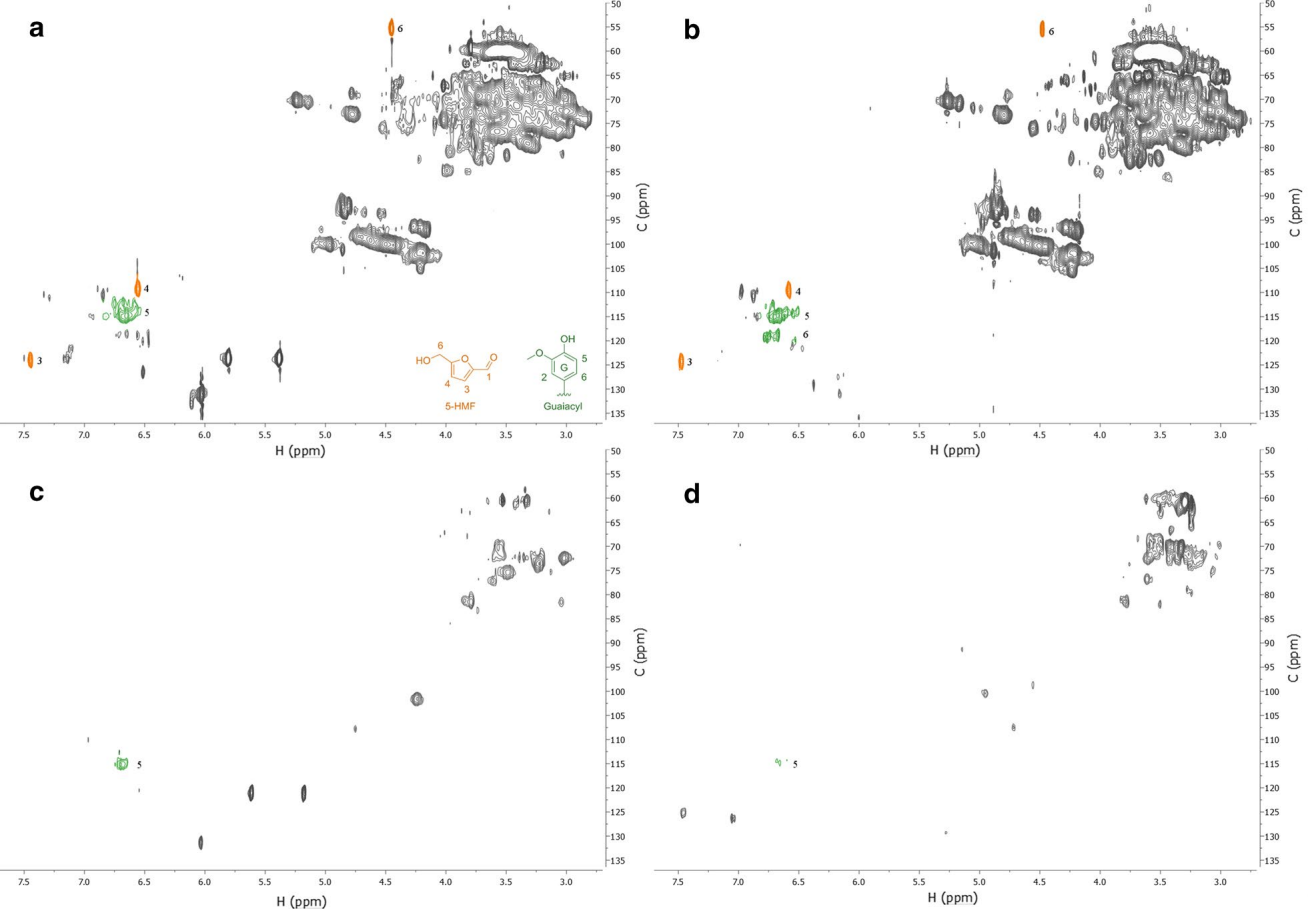
HSQC 2D NMR spectra of lignin content in biomass residues: **a** sodium citrate pH 4.0 buffered sample, **b** no buffer control, **c** sodium citrate pH 6.0 and **d** potassium phosphate pH 7.0. 5-hydroxymethylfurfural (5-HMF) and guaiacyl are depicted in the lower right of **a**, signals are colored and numbered according to the structures they relate to

The HSQC 2D-NMR experiments taken of the solvable fraction of the samples show the proton-carbon corresponding peaks. The spectra mainly contain carbohydrate signals; however, there are detectable amounts of aromatic signals in all the samples (Fig. 7). The C_5_/H_5_-signal for guaiacyl unit (G5) at 114.9/6.7 ppm has the highest intensity in the sample citrate buffer pH 4 (Fig. 7a) and in the control (Fig. 7b), and only the control sample shows the C_6_/H_6_-signal for guaiacyl unit (G6) at 118.6/6.7. Control sample (Fig. 7a) had a pH of 3.7 after steam explosion. Both the control (Fig. 7a) and citrate pH 4.0 (Fig. 7b) samples were steam exploded at a lower pH [3, 4], and the degradation of lignocellulose is more intense for both in comparison with the higher pH steam exploded samples, citrate pH 6 (Fig. 7c) and phosphate pH 7 (Fig. 7d). During SE, the lignin undergoes hydrolysis and degrades into smaller units of lignin [28]. These units should be detectable in the solvable fraction if they are small enough. With a lower pH, as in sample A and B the hydrolysis is more extensive and lignin was detected in the solvable fraction (Fig. 7). In addition to the signals from degraded lignin, there were some signals from dehydrated carbohydrates in the form of 5-hydroxymethylfurfural (5-HMF, [29]), and these were again only visible in citrate buffered sample pH 4 (A) and in the control (B) (Table 2). The pH is therefore important for control of both lignin and carbohydrate degradation.

**Table 2:** Determination of the ^13^C/^1^H correlation signals acquired in 2D-NMR HSQC of the samples and semiquantitative analysis of lignin

Label	*δ*_C_/*δ*_H_ (ppm)	Assignment	A (citrate pH 4) (%)	B (control) (%)
G5	114.9/6.7	C_5_/H_5_ in a guaiacyl unit	66	22
G6	118.6/6.7	C_6_/H_6_ in a guaiacyl unit	–	12
F3	124.1/7.5	C_3_/H_3_ in a 5-HMF unit	28	8
F4	109.4/6.6	C_4_/H_4_ in a 5-HMF unit	41	9
F6	55.4/4.5	C_6_/H_6_ in a 5-HMF unit	− 42	− 9

Based on the summarized integrated areas of 5-HMF and guaiacyl relative to co-extracted mannose, signals are calculated per 100 mannose C1/H1 signal (%)

The signals for 5-HMF (F) and guaiacyl (G) unit were integrated in NMR with the C_1_/H_1_ signal of mannose as an internal reference signal [30]. In the citrate buffer pH 4 sample, the G5-signals were 66% (calculated per 100 mannose C_1_/H_1_, Table 2) in comparison with control which had only 22%. This means that the relative amount of lignin is higher in the citrate buffer than in the control, even though the final pH in control sample was lower, as expected based on existing research [31]. As the initial pH in the control was not 3.7 before SE, the degree of hydrolysis seems to be more severe with continuously low pH. The same effect of more severe degradation is also detected with the carbohydrate fraction, as there is more 5-HMF, a common decomposition product of hexoses [32], in citric buffer (A) than control (B) (Table 2). Besides the effect on properties of extracted oligosaccharides, inhibition of polysaccharide autohydrolysis in samples soaked with buffers > 5.0 prevented the formation of HMF.

### Optimal pH range for the production of acetylated galactoglucomannan

From the wide range of combined severities tested in this study, between $R^{\prime}_{0}$ = 1.707 and $R^{\prime}_{0}$ = 0.092 (controls, citrate pH 4.0 and 5.0 buffered samples) appears to be the best range for production of acetylated galactoglucomannan. Extracts within this range contained acetylated oligosaccharides with varying DP, DA, and Man/Glc/Gal ratios. At the same time, only the control and citrate pH 4.0 samples contained detectable levels of HMF. In the range between unbuffered and pH 5.0, buffering can mitigate the deacetylation, autohydrolysis and formation of HMF, at the cost of yield. As seen in the comparison between the control sample and citrate pH 4.0, the apparent loss in yield is partly due to increased specificity toward mannan extraction. Some general trends are apparent in the data presented here: increased combined severity results in higher yields and higher degree of acetylation of extracted oligosaccharides, while at the same time reducing the degree of polymerization. Further experiments into steam explosion production of tailored oligosaccharides from Norway spruce should be focused on this severity range.

## Conclusions

Introducing buffers to a steam explosion reaction has shown to be an efficient approach for mitigating the severity of the treatment and production of a wide range of oligosaccharides from the same feedstock at the same temperature and pressure. Vast differences in monosaccharide composition, oligosaccharide size and degree of acetylation of the solubilized carbohydrate fraction were caused by the difference in pH. Notably, higher pH resulted in more pronounced deacetylation of residual biomass and extracted oligosaccharides.

Altering the pH did not reduce the severity by preventing the acetate release from the biomass, but by limiting acid hydrolysis of hemicellulose. Buffering mitigates the reactivity of acetate once it is released. The results show that the role of temperature and pressure is mainly to create conditions where autohydrolysis can occur. When the autohydrolysis was inhibited by buffering, the yields dropped and the breakdown of oligosaccharides were reduced. This study clearly shows that pH largely affects product composition and yields. It has been argued that pH has more impact on SE [12] reactions than temperature or pressure, and the results presented here support this claim.

## Materials and methods

### Buffers

1 M sodium citrate and 2 M potassium phosphate buffers were prepared by mixing 1 M solutions of sodium citrate (Sigma-Aldrich, Germany) and citric acid (Sigma-Aldrich, Germany), and 2 M solutions of di- and mono-basic potassium phosphate (Sigma-Aldrich, Germany) were mixed to reach the desired pH. Citrate buffers produced were pH 4.0, 5.0 and 6.0, and phosphate pH was 6.0, 6.5 and 7.0. The higher concentration of phosphate buffers was used to counteract the poor pH retention after SE in the phosphate buffered samples observed in initial trial experiments (unpublished).

### Wood

Dry Norway spruce (*Picea abies*) wood was milled using a hammer mill with a 2-mm sieve. 500 g samples of spruce chips was soaked with buffers and MilliQ water in a 1:1:1 (g/mL/mL) ratio prior to SE. Water was added to ensure the buffers were thoroughly mixed into the wood, resulting in final buffer concentrations of 0.5 M for sodium citrate and 1 M for potassium phosphate. The wood chips were stirred until the sample was thoroughly soaked and transferred into the SE reactor.

### Steam explosion and extraction of water soluble material

Soaked spruce chips were hydrothermally treated in a steam explosion unit (Cambi, Asker, Norway) consisting of a 20-L pressure vessel and a flash tank with collection bucket. Steam was generated in a 25 kW electric boiler (Parat, Flekkefjord, Norway). The steam explosion unit is described in detail in [33]. Treatment conditions were 200 °C, 14.5 bar; biomass residence time was 10 min.

### Handling of extracts and residuals

After SE, water was added; the slurry was stirred for extraction and filtered through a Whatman B1 filter paper (Sigma-Aldrich, Norway). The residual water insoluble material was squeezed to release the remaining soluble oligosaccharides, which were combined with the extract. Aliquots were frozen to determine extract yield and to supply samples for carbohydrate, lignin and acetyl analysis. 200 mL of each sample was freeze-dried for the analysis of constituent neutral monosaccharides (GC) and uronic acid (colorimetry) of the released oligosaccharides. Insoluble materials were dried in an oven at 100 °C for 36–48 h, to constant weight, and then milled on a cutter mill (Retsch, Haan, Germany) with a 0.5-mm sieve.

### Poly- and oligosaccharide constituent sugars, carbohydrate content and reducing sugar in extract and non-soluble residuals

Concentration of carbohydrates in solution was quantified according to the Dubois method [20] and reducing sugars content according to the Miller method [21]. Calibration curves for both colorimetric methods were based on glucose. Constituent monosaccharide of residuals and extracts were quantified by GC via alditol acetates after acid hydrolysis [34] and uronic acids in the hydrolysates were determined by a colorimetric assay [35].

### MALDI-ToF analysis

MALDI-ToF analysis of hydrolysis product was conducted on an UltraFlextreme MALDI-ToF instrument (Bruker Daltonics GmbH, Germany) equipped with a nitrogen 337-nm laser beam. Samples were prepared by applying 2 μL of a 9 mg/mL solution of 2,5-dihydroxybenzoic acid (Sigma-Aldrich, Germany) in 30% acetonitrile (VWR) to an MTP 384 ground steel target plate (Bruker Daltonics GmbH, Germany), adding 1 µL of sample (0.1–1 mg/mL) and mixing the drop with the pipette. Sample drops were then dried under a stream of warm air.

### Acetate content analysis

For the analysis of free acetate content in solution, the filtered liquid fraction washed from the biomass was diluted 1:2 with MilliQ water (to measure acetate in solution) or 100 mM KOH (to release the acetate bound to the oligosaccharides). 50 µL samples of the liquid phase was collected and analyzed by HPLC. All values were corrected for the concentration of oligosaccharides in solution and exact weight of biomass in the sample.

For the analysis of acetate content in the biomass residue, 100 mg ± 10% samples of the dried, milled residue were soaked overnight with 500 µL of 0.1 M KOH, left in a thermomixer (Eppendorf, Oslo, Norway) overnight at 1000 rpm, 40 °C. After 18 h, 500 µL of MilliQ water was added to the samples, which were then mixed by vortexing and spun down at 10,000×*g*, for 5 min, and analyzed by HPLC. All values were corrected for the concentration of oligosaccharides in solution and exact weight of biomass in the sample.

### HPLC

Acetate content was analyzed by HPLC using a REZEX ROA-Organic Acid H+ (Phenomenex, Torrance, California, USA) 300 × 7.8 mm ion exclusion column, isocratic elution with 0.6 mL/min 4 mM H_2_SO_4_ at 65 °C and UV detection at 210 nm.

### Enzymatic treatment

Milled, dry samples were washed with water to remove remaining soluble carbohydrates and buffer salts from the SE slurry, dried and resuspended in 25 mL of 50 mM sodium acetate buffer at pH 5.5. A GH5 family mannanase from *Aspergillus nidulans* [26] was applied to the sample with loadings of 0.01 mg/mg (1%), 0.1 mg/mg (10%) and 0.3 mg/mg (30%) of enzyme/mannan in samples, based on an estimate of 20% of the substrate being mannan. Samples were left in a shaking incubator overnight at 50 °C, which is the optimum temperature for enzymatic activity.

### NMR of lignin fraction

The NMR spectra were recorded on a Bruker Ascend 400 spectrometer (400 MHz) at 320 K using a 5-mm PABBO probe. The samples (45 mg) were dissolved in DMSO-d_6_ (1 mL), sonicated for 30 min and filtered through glass wool to remove any undissolved particles directly into the NMR-tube. Two of the samples did not fully dissolve. The Heteronuclear Single Quantum Coherence (HSQC) spectroscopy recorded with a spectral width of 0–12 ppm and 0–250 ppm in ^1^H and ^13^C, respectively. The number of scans for both was 512 at 27 °C. For the ^1^H-^13^C parameters, the relaxation time was 1.5 s and the free induction decay dimensions was 2048 and 256, while the number of scans were 120 at 27 °C. Integrations were done with MestReNova (version 9.1.0), where the C1 of mannose was used as an internal reference.

## Additional files

**Additional file 1: Figure S1.** 2D-NMR HSQC of Norway spruce lignin extracted by milled wood lignin [36] (MWL) method was run as a reference standard, focused on ^1^H: 2.7–7.7 and ^13^C: 50.0–135.4 ppm.

**Additional file 2: Figure S2.** MALDI-ToF spectra of extracted oligosaccharides samples from all treatment. Relative intensities show the most prevalent oligosaccharide sizes to be in the 1000–1500 *m/z* range (DP6-DP9 for hexoses) and highly acetylated in the control and citrate pH 4.0 samples. In further treatments the hexose peaks are gradually replaced with xylooligosaccharide peaks at much higher intensities and with no acetylations. The peak at 723 *m/z* is a persistent contamination.

**Additional file 3: Table S1.** Determination of the ^13^C/^1^H correlation signals acquired in the 2D-NMR HSQC spectrum of MWL spruce. β-*O*-4 reflect the β-aryl ether as sketched in Additional file 1: Figure S1, G2, -5 and -6-reflects the aromatic signals and MeO reflects the methoxyl-group in the guaiacyl units sketched in Additional file 1: Figure S1. **Table S2.** Monosaccharide composition of carbohydrates in freeze dried aliquots of filtered, water soluble fractions. **Table S3.** Monosaccharide composition of dried residual biomass. **Table S4.** Acetate present in filtered slurry and released from solubilized carbohydrates in KOH treatment. Data from Fig. 2b, c and d.
