# Supplementary figures and images for: Effects of pH on steam explosion extraction of acetylated galactoglucomannan from Norway spruce
Source: Biotechnol Biofuels. 2018 Nov 9;11:311. doi: 10.1186/s13068-018-1300-z (PMC6225635; doi:10.1186/s13068-018-1300-z)

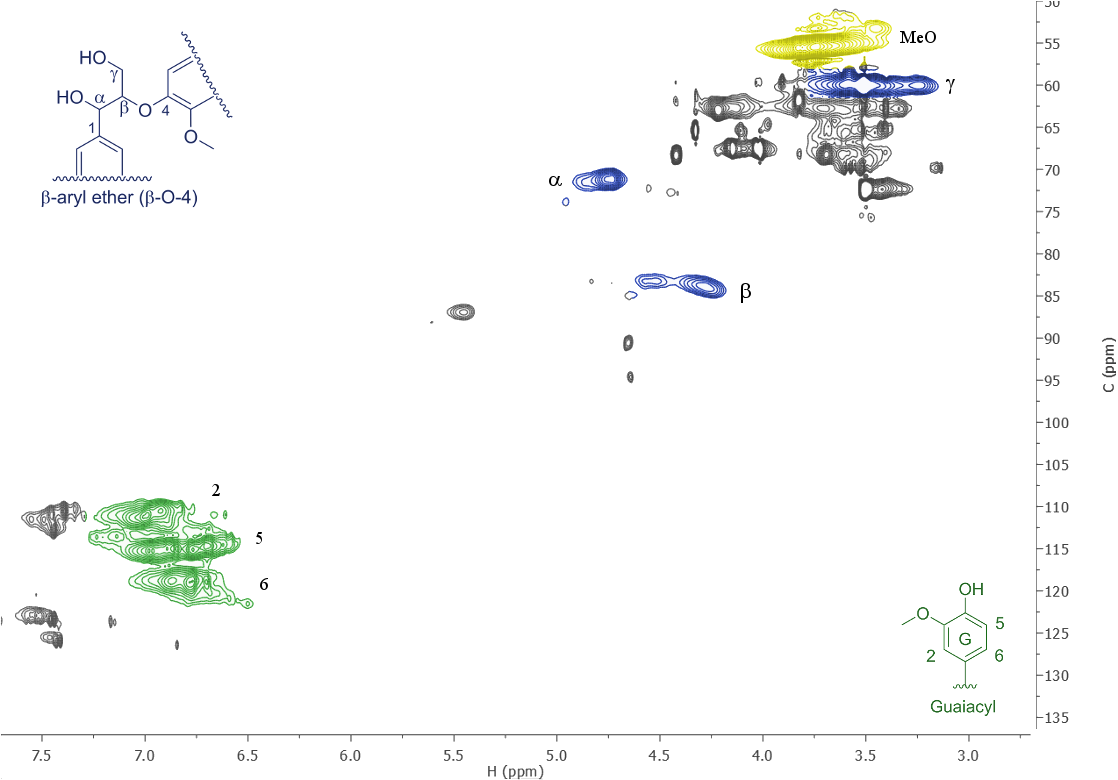

Supplement: Supplementary file 1 — Additional file 1: Figure S1. 2D-NMR HSQC of Norway spruce lignin extracted by milled wood lignin [36] (MWL) method was run as a reference standard, focused on 1H: 2.7–7.7 and 13C: 50.0–135.4 ppm. [file 13068_2018_1300_MOESM1_ESM.tif]

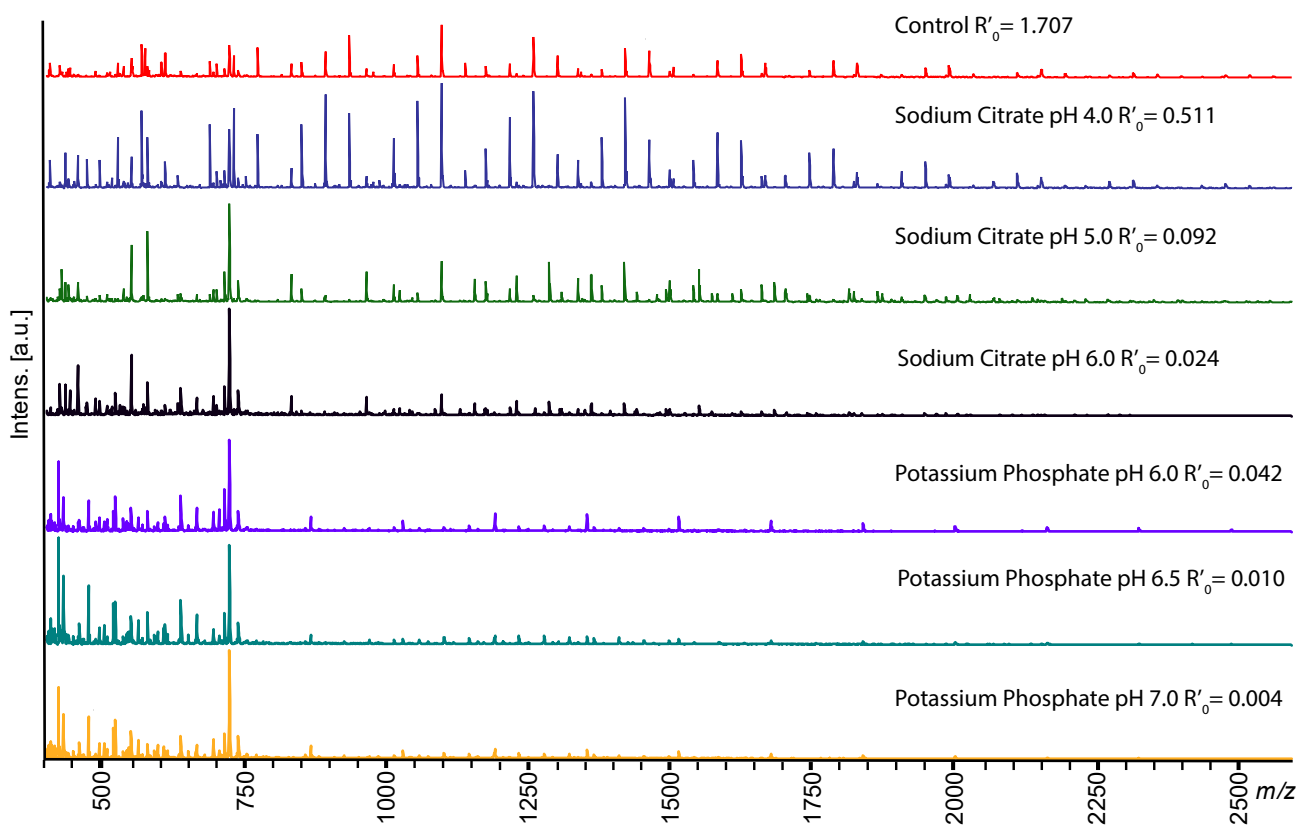

Supplement: Supplementary file 2 — Additional file 2: Figure S2. MALDI-ToF spectra of extracted oligosaccharides samples from all treatment. Relative intensities show the most prevalent oligosaccharide sizes to be in the 1000–1500 m/z range (DP6-DP9 for hexoses) and highly acetylated in the control and citrate pH 4.0 samples. In further treatments the hexose peaks are gradually replaced with xylooligosaccharide peaks at much higher intensities and with no acetylations. The peak at 723 m/z is a persistent contamination. [file 13068_2018_1300_MOESM2_ESM.pdf]
