# Supplementary material for: Effects of pH on steam explosion extraction of acetylated galactoglucomannan from Norway spruce
Source: Biotechnol Biofuels. 2018 Nov 9;11:311. doi: 10.1186/s13068-018-1300-z (PMC6225635; doi:10.1186/s13068-018-1300-z)
Supplement: Supplementary file 3 — Additional file 3: Table S1. Determination of the 13C/1H correlation signals acquired in the 2D-NMR HSQC spectrum of MWL spruce. β-O-4 reflect the β-aryl ether as sketched in Additional file 1: Figure S1, G2, -5 and -6-reflects the aromatic signals and MeO reflects the methoxyl-group in the guaiacyl units sketched in Additional file 1: Figure S1. Table S2. Monosaccharide composition of carbohydrates in freeze dried aliquots of filtered, water soluble fractions. Table S3. Monosaccharide composition of dried residual biomass. Table S4. Acetate present in filtered slurry and released from solubilized carbohydrates in KOH treatment. Data from Fig. 2b, c and d. [file 13068_2018_1300_MOESM3_ESM.docx]

Table S1. Determination of the ^13^C/^1^H correlation signals acquired in the 2D-NMR HSQC spectrum of MWL spruce.β-O-4 reflect the β-aryl ether as sketched in figure S1, G2, -5 and -6-reflects the aromatic signals and MeO reflects the methoxyl-group in the guaiacyl units sketched in figure S1.

| Label | δ_C_/δ_H_ (ppm) | Assignment |
| --- | --- | --- |
| MeO | 55.7/3.7 | C/H is methoxyl- in C_3_ position on a guaiacyl unit |
| β–O–4α | 71.9/4.8 | C_α_/H_α_ from β–O–4 lignin bonding pattern |
| β–O–4β | 86.1/4.1 | C_β_/H_β_ from β–O–4 lignin bonding pattern |
| β–O–4γ | 59.7/3.5 | C_γ_/H_γ_ from β–O–4 lignin bonding pattern |
| G2 | 111.0/7.0 | C_2_/H_2_ in a Guaiacyl unit |
| G5 | 114.9/6.8 | C_5_/H_5_ in a Guaiacyl unit |
| G6 | 118.9/6.8 | C_6_/H_6_ in a Guaiacyl unit |

Table S2. Monosaccharide composition of carbohydrates in freeze dried aliquots of filtered, water soluble fractions.

| Sample: | Combined severity R’_0:_ | Rha | Fuc | Ara | Xyl | Man | Gal | Glc |
| --- | --- | --- | --- | --- | --- | --- | --- | --- |
| Control | 1.707 | 0.90 | n.d. | 4.95 | 15.94 | 46.41 | 7.10 | 24.68 |
| Citrate pH 4.0 | 0.511 | 0.95 | n.d. | 2.97 | 14.30 | 54.02 | 7.87 | 19.89 |
| Citrate pH 5.0 | 0.092 | 1.60 | n.d. | 7.82 | 28.09 | 32.55 | 10.17 | 19.78 |
| Phosphate 6.0 | 0.042 | 0.75 | n.d. | 5.29 | 17.27 | 40.59 | 6.95 | 29.14 |
| Citrate pH 6.0 | 0.024 | 2.22 | n.d. | 12.72 | 41.35 | 13.40 | 10.52 | 19.79 |
| Phosphate pH 6.5 | 0.010 | 0.63 | n.d. | 6.99 | 23.38 | 10.10 | 6.11 | 52.79 |
| Phosphate 7.0 | 0.004 | 1.08 | n.d. | 8.06 | 27.49 | 10.47 | 7.16 | 45.75 |

Table S3. Monosaccharide composition of dried residual biomass.

| Sample: | Combined severity R’_0:_ | Rha | Fuc | Ara | Xyl | Man | Gal | Glc |
| --- | --- | --- | --- | --- | --- | --- | --- | --- |
| Control | 1.707 | n.d. | n.d. | n.d. | 22.62 | 37.56 | 6.01 | 30.20 |
| Citrate pH 4.0 | 0.511 | n.d. | n.d. | 0.94 | 22.09 | 39.51 | 6.92 | 27.60 |
| Citrate pH 5.0 | 0.092 | n.d. | n.d. | 2.49 | 18.51 | 48.33 | 8.14 | 19.37 |
| Phosphate pH 6.0 | 0.042 | n.d. | n.d. | 3.28 | 17.63 | 46.15 | 7.42 | 22.16 |
| Citrate pH 6.0 | 0.024 | n.d. | n.d. | 3.58 | 18.91 | 47.62 | 7.57 | 18.84 |
| Phosphate pH 6.5 | 0.010 | n.d. | n.d. | 4.70 | 20.56 | 43.42 | 7.07 | 20.11 |
| Phosphate pH 7.0 | 0.004 | n.d. | n.d. | 5.39 | 22.36 | 41.22 | 6.90 | 19.48 |
| Wood | N/A | 3.90 | 0.00 | 1.95 | 8.72 | 19.89 | 2.68 | 66.77 |

Table S4. Acetate present in filtered slurry and released from solubilized carbohydrates in KOH treatment. Data from Figure 2B,C and D.

| Combined severity R’_0_: | Acetate in solution in µmole/mg carbohydrate. | Alkali released acetate in  µmole/mg carbohydrate. | Acetate in biomass residue in µmole/mg biomass. |
| --- | --- | --- | --- |
| 1.707 | 0.08 | 0.30 | 0.14 |
| 0.511 | 0.40 | 0.30 | 0.06 |
| 0.092 | 1.13 | 0.11 | 0.06 |
| 0.042 | 1.71 | n.d. | 0.06 |
| 0.024 | 2.20 | n.d. | 0.07 |
| 0.010 | 2.33 | n.d. | 0.05 |
| 0.004 | 2.64 | n.d. | 0.08 |
| Wood | - | - | 0.22 |
